# Use of Complex DNA and Antibody Microarrays as Tools in Functional Analyses

**DOI:** 10.1002/cfg.320

**Published:** 2003-10

**Authors:** Andrea Bauer, Boris Beckmann, Christian Busold, Ole Brandt, Wlad Kusnezow, Janne Pullat, Verena Aign, Kurt Fellenberg, Robert Fleischer, Anette Jacob, Marcus Frohme, Jörg D. Hoheisel

**Affiliations:** Division of Functional Genome Analysis Deutsches Krebsforschungszentrum (DKFZ) Im Neuenheimer Feld 580 Heidelberg D-69120 Germany

## Abstract

While the deciphering of basic sequence information on a genomic scale is yielding complete genomic sequences in ever-shorter intervals, experimental procedures for elucidating the cellular effects and consequences of the DNA-encoded information become critical for further analyses. In recent years, DNA microarray technology has emerged as a prime candidate for the performance of many such functional assays. Technically, array technology has come a long way since its conception some 15 years ago, initially designed as a means for large-scale mapping and sequencing.

The basic arrangement, however, could be adapted readily to serve eventually as an analytical tool in a large variety of applications. On their own or in combination with other methods, microarrays open up many new avenues of functional analysis.


**Comparative and Functional Genomics**
*Comp Funct Genom* 2003; **4**: 520–524.
Published online in Wiley InterScience (www.interscience.wiley.com). **DOI:** 10.1002/cfg.320

Conference Review

### Use of complex DNA and antibody microarrays as tools in functional analyses

Andrea Bauer, Boris Beckmann, Christian Busold, Ole Brandt, Wlad Kusnezow, Janne Pullat, Verena Aign, Kurt Fellenberg, Robert Fleischer, Anette Jacob, Marcus Frohme and Jörg D. Hoheisel*
*Division of Functional Genome Analysis, Deutsches Krebsforschungszentrum (DKFZ), Im Neuenheimer Feld 580, D-69120 Heidelberg, Germany*

**Correspondence to: Jörg D. Hoheisel, Division of Functional Genome Analysis, Deutsches Krebsforschungszentrum (DKFZ), Im Neuenheimer Feld 580, D-69120 Heidelberg, Germany. E-mail: j.hoheisel@dkfz.de*

Received: 29 July 2003
Revised: 3 August 2003
Accepted: 4 August 2003

### Abstract

**While the deciphering of basic sequence information on a genomic scale is yielding complete genomic sequences in ever-shorter intervals, experimental procedures for elucidating the cellular effects and consequences of the DNA-encoded information become critical for further analyses. In recent years, DNA microarray technology has emerged as a prime candidate for the performance of many such functional assays. Technically, array technology has come a long way since its conception some 15 years ago, initially designed as a means for large-scale mapping and sequencing. The basic arrangement, however, could be adapted readily to serve eventually as an analytical tool in a large variety of applications. On their own or in combination with other methods, microarrays open up many new avenues of functional analysis.**
**Copyright © 2003 John Wiley & Sons, Ltd.**

In the DKFZ Division of Functional Genome Analysis, we are developing technologies for the identification, description and evaluation of cellular functions and their regulation, by producing and processing biological information on a genomic scale. One emphasis in our effort is work on DNA, protein and peptide microarrays. Many chemical and biophysical issues are being addressed in an attempt to understand the underlying procedural aspects, thereby eventually establishing superior analysis procedures. Based on technical advances, the resulting methods are immediately put to the test in relevant, biologically-driven studies on various organisms. Concerning the analysis of human material, systems are being developed toward early diagnosis, prognosis and evaluation of the success of disease treatment, with an emphasis on cancer.

### Determination of base variations

Genotyping has developed enormously with the advent of single nucleotide polymorphisms (SNPs). Not only are there very many of them but they can also be assayed in a parallel format. Nevertheless, the capacity for large-scale analyses is still critical to many potential applications. Also, the selection of informative SNPs is a limiting aspect. We are performing SNP-typing experiments in molecular epidemiological studies, for example. In collaboration with Alexandra Nieters and Nikolaus Becker (DKFZ, Heidelberg), a microarray has been established for the analysis of SNPs associated with the existence of lymphoid neoplasms (Figure 1). The initial aim of this project is a combination of epidemiological data obtained from a case-control study of about 600 patients and 600 matched unaffected individuals with molecular information on some 100 appropriate SNPs. This study is part of a much wider effort coordinated by an international network called Interlymph (**http://dceg2.cancer.gov/newsletter/News0303.html#interlymph**), in which some 20 000 case and control samples await genetic analysis. We are using the Geniom® platform of febit (Mannheim, Germany) for the generation and use of complex oligonucleotide arrays. The device currently permits *in situ* synthesis of microarrays that contain up to 64 000 different oligonucleotides. All of the steps

Copyright © 2003 John Wiley & Sons, Ltd.

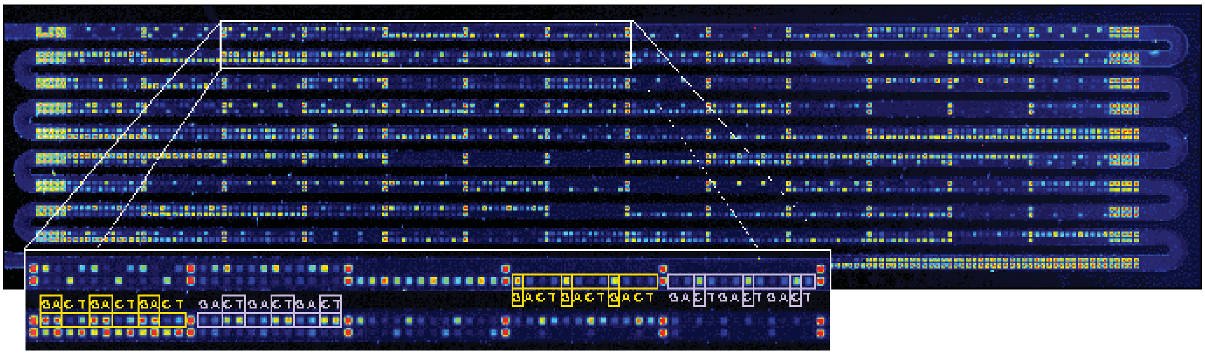

**Figure 1.** SNP-typing on an oligonucleotide microarray. The microarray was produced using the Geniom® unit from febit (Mannheim, Germany). The oligonucleotides had a length of 20 nt. Only a portion of the entire microarray is shown. A simultaneous hybridization of PCR products that represented 95 SNPs yielded the binding pattern that is presented. Examples of heterozygous and homozygous polymorphisms are highlighted, which were also confirmed by enzymatic sequencing necessary for *in situ* oligomer synthesis (starting from an empty cartridge), sample hybridization and detection are carried out within the device and on site. Any combination of oligonucleotide sequences can be generated on the microarray, based on individual data files created or assembled by the user. Therefore, empirical results from earlier hybridizations can immediately be applied to the improvement of the next microarray. In another collaborative effort (with Ethel de Villiers, also at DKFZ), the system is used to establish an array-based assay for the identification and discrimination of all Human Papilloma Virus (HPV) types by virtue of differences in their sequence. Some HPV types are considered to be necessary aetiological factors for the development of cervical cancer, while others are mainly associated with benign lesions. A third form of analysis — done in collaboration with Frank Lyko (DKFZ) and the company Epigenomics (Berlin, Germany) — aims at the elucidation of the methylation status of genomic DNA. For this kind of study, genomic DNA is treated with bisulphite. While methylated cytosine remains unaffected, unmethylated cytosine is chemically transformed into uracil, and subsequently thymidine upon PCR amplification. This transformation can be assayed as a polymorphism at the respective site by comparing DNA before and after bisulphite treatment [1].

With the availability of photolabile 3′-*O*-[2(2-nitrophenyl)propoxycarbonyl]-protected 5′-phosphoramidites [2], an alternative mode of light-directed production of oligonucleotide arrays became possible. Because of the characteristics of these building blocks, light-controlled *in situ* DNA synthesis occurs in the 5′–3′ direction, conforming to the orientation of enzymatic synthesis. Thus, the 3′-termini of the eventual oligonucleotides can act as substrates for on-chip polymerase reactions. The production of such oligonucleotide arrays adds new procedural avenues to DNA microarrays. With respect to genotyping, complexity of the samples, and thus throughput, can be increased substantially [3].

Even further advances could be expected by using a process that circumvents the fact that the DNA samples to be studied usually require (PCR) amplification and (fluorescence) labelling prior to analysis. The structural difference between peptide nucleic acid (PNA) — used as probe molecule on the array — and a DNA target permits direct detection of a binding event. Upon hybridization of a nucleic acid sample to a PNA array, the phosphates of the DNA/RNA can be utilized as an intrinsic label for detection by secondary ion mass spectrometry (SIMS); PNA molecules are lacking phosphate groups entirely. In collaboration with Heinrich Arlinghaus (University of Münster), we established the basic processes for analysing DNA by such means [4,5].

### Transcriptional profiling

For understanding the complex regulative mechanisms and investigating the management of cellular

Copyright © 2003 John Wiley & Sons, Ltd. 

control, a parallel determination of the expression of all of the genes of an organism or tissue is indispensable. One of many practical applications pursued by us is the analysis of pancreatic tumourigenesis. Pancreatic cancer is the fifth most common cause of cancer-related deaths in industrialized countries, with a dismal prognosis, an increasing incidence and no, or only rather ineffective, means of treatment. The development of new treatment modalities and diagnostic and preventive approaches requires an understanding of the molecular mechanisms of tumourigenesis in the pancreas. In collaboration with Thomas Gress (University of Ulm, Germany) and Helmut Friess (University of Heidelberg, Germany), we are analysing a set of genes known to be specifically differentially transcribed in pancreatic tumours in clinical samples. Towards the objective of performing diagnosis — on the basis of which eventually prognosis might be possible — or for the identification of potential target molecules, the selection of appropriate probes and the availability of good-quality tissue samples are essential, but nevertheless only initial, steps. Just as important is data integration, since tying connections between molecular and clinical data makes subsequent interpretation more likely to succeed. This process requires a modular data warehouse concept, in which experimental data, such as raw signal intensities or gene annotations, are stored in combination with the clinical information available on the samples/patients in a pre-defined and catalogued vocabulary ([6,7]; **www.dkfz-heidelberg.de/funct_genome/index.html#mchips;** currently a total of 4755 hybridization experiments are stored). Then, statistical algorithms can be utilized for the identification of molecular factors that are characteristic for a certain subgroup of samples, and thus patients, for example (Figure 2). In addition, the association

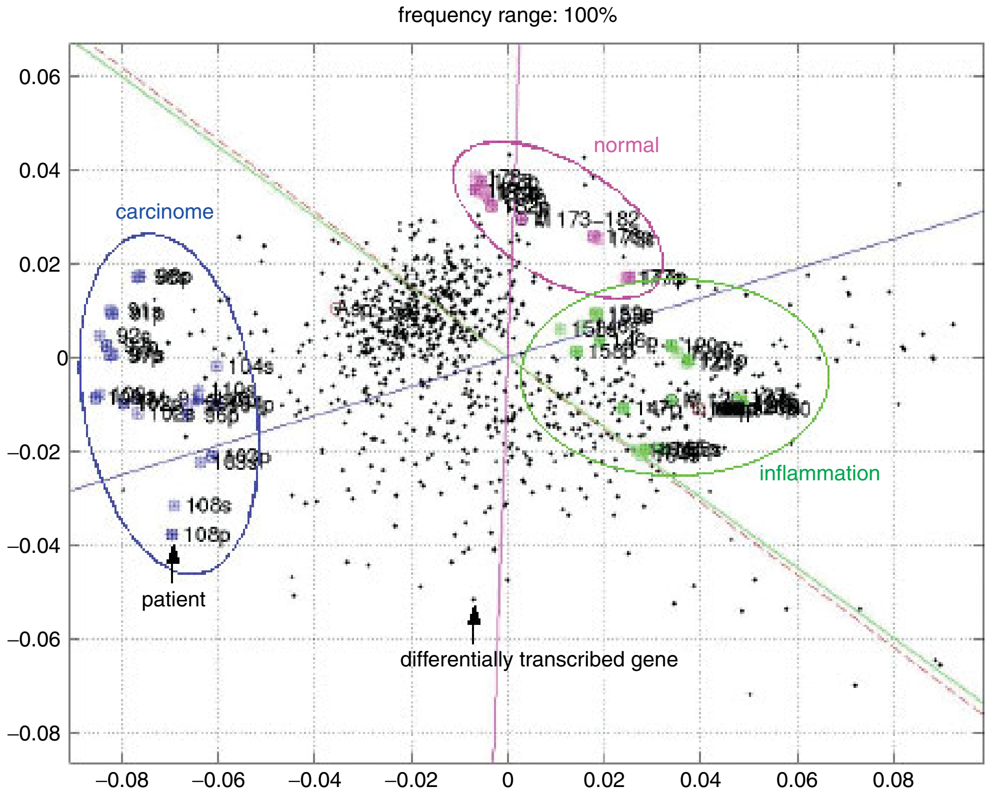

**Figure 2.** Biplot presentation of the results of a correspondence analysis [7]. Transcriptional profiling data were generated from normal pancreatic tissues, material from patients with chronic pancreatitis, and cancer patients. The various samples could be discriminated easily, forming distinct clusters that — expectedly — have a very high correlation with the origin of the various samples

Copyright © 2003 John Wiley & Sons, Ltd. 

of groups of genes with a certain type of disease progression, or their — potentially even indirect — relevance to a particular pathway, may be identified in a more automated fashion.

For many applications, the availability of microarrays that represent the entire genome rather than the coding sequences only would be beneficial. Such an array would, by definition, contain all of the genes of the given organism, irrespective of the status and quality of sequence annotation. More importantly, all genomic regions, including the important regulatory portions, for example, would become accessible to analysis. Along these lines, we have produced a minimal tiling path of fragments from the shotgun clones used for sequencing the genome of *Pseudomonas putida* [8], for instance. Further work in this direction is in progress.

### Protein expression

While two-dimensional gel electrophoresis does provide a powerful technique for the analysis of at least a large number of proteins of an organism or tissue, many other powerful methods are nevertheless prerequisite to approaching the world of protein analysis in a manner similar to what is already possible for studies at the level of nucleic acids [9,10]. However, the biochemical diversity and the sheer number of proteins are such that an equivalent analysis is much more complex and thus difficult to accomplish. Performing microarray immunoassays, for instance, represents a challenge even at the level of preparing a working microarray surface. We compared different strategies for producing antibody microarrays on glass slides, analysing the effect of multiple factors — the modification of the glass surface, the kind and length of cross-linkers, the composition and pH of the spotting buffer, blocking reagents, antibody concentration and storage procedures — on array performance. Data from nearly 1000 slides were analysed for this evaluation, and to establish appropriate assay conditions [11,12].

Initial experiments aimed at an actual expression profiling of cancer tissues are under way. On the basis of transcriptional profiling experiments using a microarray that contains thousands of cancer-associated genes, several hundred differentially transcribed genes of interest were selected. Antibodies for their respective proteins were generated in cooperation with the company Eurogentec (Seraing, Belgium) by synthesizing sequence-specific peptides, which in turn were used for immunization of rabbits. This process provided us with an initial set of antibodies that are being used as probes on antibody microarrays. For more global studies, other selection methods and antibodies that originate from recombinant antibody libraries will be required. Comparative analyses of protein expression and transcriptional changes observed in the same tissues are under way.

### Conclusion

Although microarray technology has grown from infancy into a lively teenager, one should keep in mind nevertheless that there is much more to come from this type of analysis and that the technique's current behaviour is not always at its best. Nevertheless, its contribution to functionally oriented research is already tremendous, although still dwarfed by the potential that can be tapped into. The range in the status of current developments is wide. While initial assays are already entering the diagnostic market, new areas of application are still being developed, and await exploitation.

### Acknowledgements

The authors wish to thank many colleagues for valuable discussions and technical support. Work in this laboratory was funded by the German Federal Ministry of Education and Research (BMBF), the European Commission, Deutsche Krebshilfe and the Deutsche Forschungsgemeinschaft (DFG).

### References

1. Adorjan P, Distler J, Lipscher E, *et al.* 2002. Tumour class prediction and discovery by microarray-based DNA methylation analysis. *Nucleic Acids Res* **30**: e21.
2. Beier M, Stephan A, Hoheisel JD. 2001. Synthesis of photolabile 5′-O-phosphoramidites for the production of microarrays of inversely oriented oligonucleotides. *Helvet Chim Acta* **84**: 2089–2095.
3. Tonisson N, Zernant J, Kurg A, *et al.* 2002. Evaluating the arrayed primer extension resequencing assay of TP53 tumor suppressor gene. *Proc Natl Acad Sci USA* **99**: 5503–5508.

Copyright © 2003 John Wiley & Sons, Ltd. 

4. Arlinghaus HF, Ostrop M, Friedrichs O, Feldner J. 2003. Genome diagnostic with TOF–SIMS. *Appl Surf Sci* **203/204**: 689–692.
5. Jacob A, Brandt O, Würtz S, *et al*. 2003. Production of PNA-arrays for nucleic acid detection. In *PNA: A Laboratory Manual*, Nielsen PE (ed.) (in press).
6. Fellenberg K, Hauser NC, Brors B, Hoheisel JD, Vingron M. 2002. Microarray data warehouse allowing for the statistical analysis of experiment annotations. *Bioinformatics* **18**: 423–433.
7. Fellenberg K, Vingron M, Hauser NC, Hoheisel JD. 2003. Correspondence analysis with microarray data. In *Perspectives in Gene Expression*, Appasani K (ed.). Eaton: Westborough; 307–343.
8. Stjepandic D, Weinel C, Hilbert H, *et al*. 2002. The genome structure of *Pseudomonas putida*; high-resolution mapping and microarray analysis. *Environ Microbiol* **4**: 819–823.
9. Hoheisel JD, Cahill D. 2002. Proteomics and genomics; catching function in action. *Curr Opin Chem Biol* **6**: 11–12.
10. Kusnezow W, Hoheisel JD. 2002. Antibody microarrays: promises and problems. *BioTechniques* **33**(suppl.): 14–23.
11. Kusnezow W, Hoheisel JD. 2003. Solid support for microarray immunoassays. *J Mol Recognit* **16**: 165–176.
12. Kusnezow W, Jacob A, Walijew A, Diehl F, Hoheisel JD. 2003. Antibody microarrays: an evaluation of production parameters. *Proteomics* **3**: 254–264.

Copyright © 2003 John Wiley & Sons, Ltd. 
